# Diagnostic accuracy of MRI-based radiomic features for EGFR mutation status in non-small cell lung cancer patients with brain metastases: a meta-analysis

**DOI:** 10.3389/fonc.2024.1428929

**Published:** 2025-01-06

**Authors:** Yuqin Long, Rong Zhao, Xianfeng Du

**Affiliations:** ^1^ Department of Respiratory and Critical Care Medicine, The Affiliated Dazu’s Hospital of Chongqing Medical University, Chongqing, China; ^2^ Department of Radiology, The Affiliated Dazu’s Hospital of Chongqing Medical University, Chongqing, China; ^3^ Department of Oncology, The Affiliated Dazu’s Hospital of Chongqing Medical University, Chongqing, China

**Keywords:** MRI, radiomics, non-small cell lung cancer, brain metastases, EGFR mutations

## Abstract

**Objective:**

This meta-analysis aims to evaluate the diagnostic accuracy of magnetic resonance imaging (MRI) based radiomic features for predicting epidermal growth factor receptor (EGFR) mutation status in non-small cell lung cancer (NSCLC) patients with brain metastases.

**Methods:**

We systematically searched PubMed, Embase, Cochrane Library, Web of Science, Scopus, Wanfang, and China National Knowledge Infrastructure (CNKI) for studies published up to April 30, 2024. We included those studies that utilized MRI-based radiomic features to detect EGFR mutations in NSCLC patients with brain metastases. Sensitivity, specificity, positive and negative likelihood ratios (PLR, NLR), and area under the curve (AUC) were calculated to evaluate the accuracy. Quality assessment was performed using the quality assessment of prognostic accuracy studies 2 (QUADAS-2) tool. Meta-analysis was conducted using random-effects models.

**Results:**

A total of 13 studies involving 2,348 patients were included. The pooled sensitivity and specificity of MRI-based radiomic features for detecting EGFR mutations were 0.86 (95% CI: 0.74-0.93) and 0.83 (95% CI: 0.72-0.91), respectively. The PLR and NLR were calculated as 5.14 (3.09, 8.55) and 0.17 (0.10, 0.31), respectively. Substantial heterogeneity was observed, with I² values exceeding 50% for all parameters. The AUC for the receiver operating characteristic analysis was 0.91 (95% CI: 0.88-0.93). Subgroup analysis indicated that deep learning models and studies conducted in Asian showed higher diagnostic accuracy compared to their respective counterparts.

**Conclusions:**

MRI-based radiomic features demonstrate a high potential for accurately detecting EGFR mutations in NSCLC patients with brain metastases, particularly when advanced deep learning techniques were employed. However, the variability in diagnostic performance across different studies underscores the need for standardized radiomic protocols to enhance reproducibility and clinical utility.

**Systematic review registration:**

https://www.crd.york.ac.uk/prospero/, identifier CRD42024544131.

## Introduction

Lung cancer remains the leading cause of cancer-related mortality worldwide, with non-small cell lung cancer (NSCLC) accounting for approximately 85% of all cases (1). Among the various genetic aberrations associated with NSCLC, mutations in the Epidermal Growth Factor Receptor (EGFR) gene are of particular clinical importance, especially due to their implications for targeted therapies (2). EGFR mutations are predominantly observed in adenocarcinoma subtype and are more common in non-smokers and individuals of East Asian ethnicity (3). Despite advances in treatment, NSCLC patients frequently develop brain metastases, which significantly worsen their prognosis and quality of life (4). The detection of EGFR mutations in patients with brain metastases is crucial, as brain metastases may exhibit different EGFR statuses due to genetic heterogeneity, which directly affects drug selection and treatment outcomes. Accurately identifying EGFR mutations in brain metastases helps in choosing the most suitable EGFR tyrosine kinase inhibitors, which can effectively penetrate the blood-brain barrier and improve outcomes (5). However, the determination of EGFR mutation status can be challenging due to the difficulties in obtaining brain tissue samples, highlighting the need for non-invasive diagnostic methods.

Non-invasive diagnostic methods for detecting EGFR mutations in NSCLC have significantly evolved, enhancing patient management by offering safer and more accessible options. Liquid biopsy is one of the most transformative methods, enabling the detection and monitoring of EGFR mutations through circulating tumor DNA (ctDNA) in blood samples, with numerous studies highlighting its clinical utility and concordance with tissue-based genotyping (6, 7). Similarly, the analysis of circulating tumor cells (CTCs) offers an alternative, albeit with varying sensitivity based on tumor burden and disease stage (8, 9). Beyond cellular analysis, advancements in molecular imaging, such as PET scans using specific tracers designed to target mutant proteins, are under investigation, potentially allowing direct visualization of EGFR mutations within tumor sites (10). Among these methods, MRI-based radiomic features stand out for their ability to non-invasively assess tumor characteristics through advanced image processing techniques. By analyzing high-throughput data extracted from standard MRI scans, radiomics allows for the detailed characterization of tumor phenotype and microenvironment, offering a promising avenue for predicting EGFR mutation status without the need for invasive procedures (11).

Recent studies have demonstrated that radiomic features extracted from MRI scans can differentiate between EGFR-mutant and wild-type tumors effectively. It was reported that specific radiomic signatures from brain MRI were significantly associated with EGFR mutation status in primary lung cancer, suggesting a potential non-invasive biomarker for clinical use (12). However, the utility of MRI radiomics in clinical practice remains limited by variability in imaging protocols, the need for robust validation in large-scale studies, and integration into clinical workflows.

Our research targeted NSCLC patients with brain metastases, utilizing MRI-based radiomic features to detect EGFR mutation status. The effectiveness was assessed through diagnostic accuracy measures such as sensitivity, specificity, positive and negative likelihood ratios (PLR, NLR), and area under the curve (AUC). Accordingly, our study aimed to establish the potential of MRI radiomics in facilitating non-invasive diagnostic processes, thereby providing more accurate treatment guidance for this patient population.

## Methods

This study was conducted using the guidance from Preferred Reporting Items for Systematic reviews and Meta-Analysis protocols (PRISMA flowchart). This study was registered with the International Prospective Register of Systematic Reviews (PROSPERO), registration number CRD42024544131.

### Systematic literature search

A comprehensive literature search was conducted using multiple electronic databases to identify relevant studies for inclusion in this meta-analysis. The databases searched included PubMed, Embase, Cochrane Library, Web of Science, Scopus, Wanfang, and China National Knowledge Infrastructure (CNKI). The search strategy utilized a combination of medical subject headings (MeSH) terms and keywords related to “non-small cell lung cancer”, “EGFR mutations”, “brain metastases”, “MRI” (**Supplementary Table S1**). The search was limited to studies published in English or Chinese languages from inception to April 30, 2024. Additionally, manual searches of reference lists from relevant articles and systematic reviews were performed to identify additional studies missed by the electronic search. Duplicate records were removed, and the remaining articles were screened based on titles and abstracts for relevance. Full-text articles of potentially eligible studies were then assessed for final inclusion based on the Inclusion and exclusion criteria.

### Inclusion and exclusion criteria

The inclusion criteria were set as follows: 1) studies published in peer-reviewed journals; 2) studies performed in patients with NSCLC and diagnosed with brain metastases; 3) studies related to the diagnostic accuracy of MRI-based radiomics features for predicting EGFR mutation status; 4) adequate data to calculate sensitivity, specificity, PLR, NLR and AUC; 5) validated methods for identification of EGFR mutation status confirmed by tissue biopsy or molecular testing, and 6) publications in English or Chinese.

Exclusion criteria were as follows: 1) conference abstracts, case reports, letters, editorials, or review articles; 2) focused solely on non-diagnostic outcomes or therapeutic interventions; 3) lacking essential data required for analysis; 4) duplicates or overlapping datasets from the same study population; and 5) not accessible in full-text format.

### Data extraction

Data extraction was performed independently by two reviewers using a standardized data extraction form. The following information was extracted from each included study: author names, country of study, study period, number of patients included, patient demographics (age and gender distribution), EGFR mutation rate, specific exons of EGFR mutations examined, MRI vendors used for scanning, imaging sequence utilized (e.g., T1-weighted, T2-weighted), segmentation method for extracting radiomic features, and modeling method employed for analysis. Any discrepancies between reviewers were resolved through discussion or consultation with a third reviewer to achieve consensus.

### Quality assessment

The methodological quality and risk of bias in diagnostic accuracy studies were assessed using the QUADAS-2 tool, designed to assess this specifically. Each included study was assessed for: patient selection, index test, reference standard, flow, and timing. We conducted assessments for each domain (including risk of bias and concerns about applicability). Studies were categorized as having low, high, or unclear risk of bias based on the assessment results (13). The objective of this process was to ascertain the credibility and validity of the studies included in the meta-analysis, reducing bias.

### Statistical analysis

Statistical analyses were performed using Stata 16.0 and MetaDisc 1.4 software. We calculated diagnostic accuracy variables: sensitivity, measuring the proportion of correctly identified patients (positive) carrying EGFR mutations; specificity, measuring the proportion of true negatives (without mutations) accurately identified; PLR, indicating the increase in disease odds with a positive test result; NLR, indicating the decrease in disease odds with a negative test result; AUC, evaluating the overall diagnostic ability of the radiomic features. Data from included studies were synthesized using random-effects models to account for study variability, with I² statistics evaluating heterogeneity (I^2^ >50% indicating substantial heterogeneity). We pooled sensitivity, specificity, PLR, NLR, and AUC with their respective 95% confidence intervals (CI) as outcome indicators. To explore sources of heterogeneity, we conducted subgroup analyses based on geographic region, segmentation strategy, and modeling method. Furthermore, the presence of publication bias was assessed by Deeks’ funnel plot asymmetry test. For all tests, statistical significance was considered p < 0.05.

## Results

### Study selection

**Figure 1:**
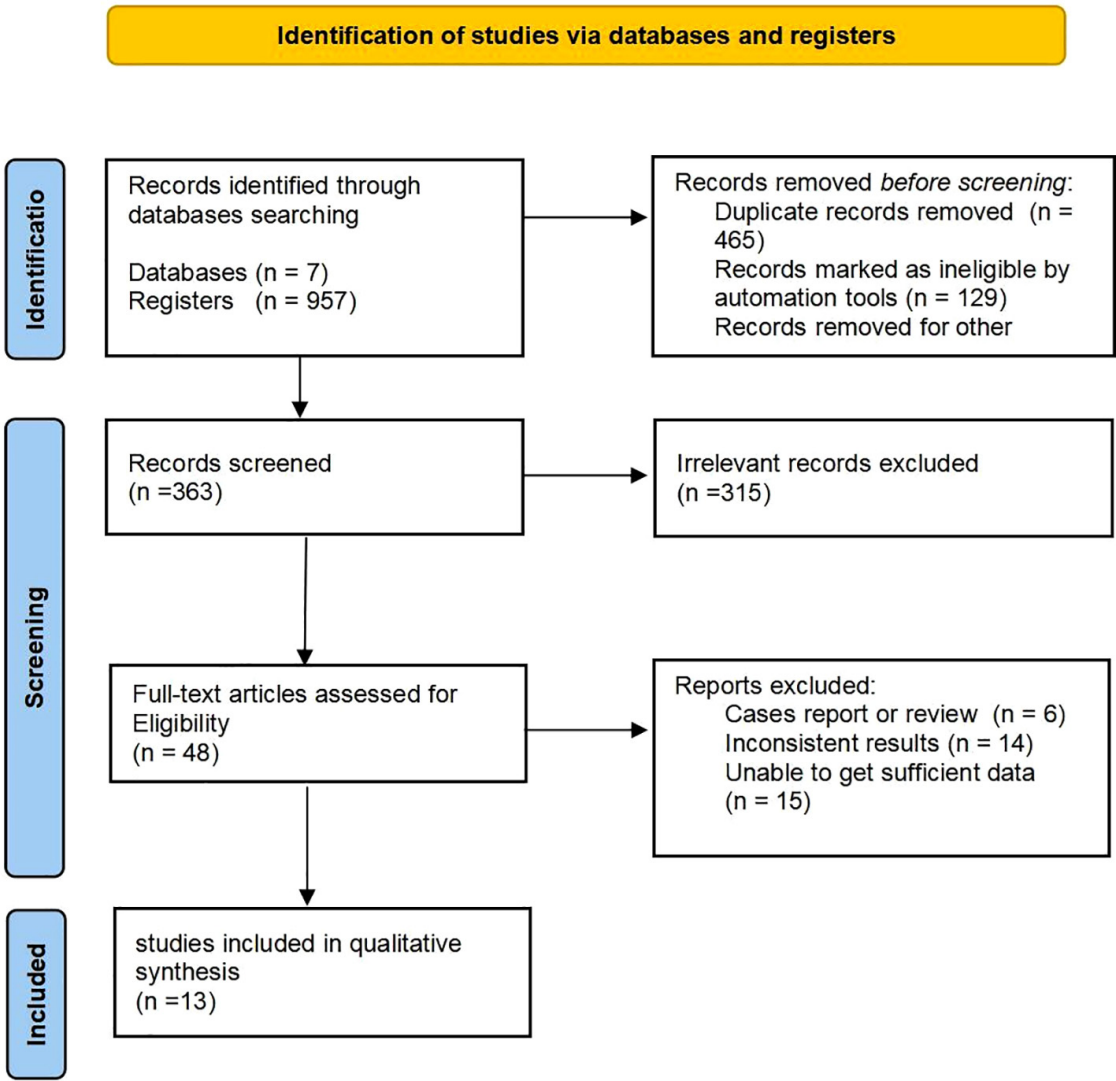
Flow diagram of study selection.

The electronic search yielded 957 potentially eligible citations. Of these, 465 were identified as duplicates and 129 were automatically disqualified. Following a evaluation of titles and abstracts, 315 articles were excluded based on the inclusion and exclusion criteria. Subsequently, reviewers conducted a detailed assessment of 48 full-text articles, ultimately selecting 13 studies for inclusion in the meta-analysis (14–26) (**Figure 1**).

### Characteristics of the enrolled studies

The characteristics of the included studies were summarized in **Table 1**. Studies were conducted in multiple countries, including China, Korea, USA, Israel, Italy, and Turkey, spanning from 2009 to 2023. The sample sizes varied widely, ranging from 30 to 405 patients, with mean patient ages ranging from 56.6 to 64.1 years. Gender distribution across studies varied, with some reporting specific numbers of male and female patients. EGFR mutation rates ranged from 26.7% to 72.2%, with different exons of EGFR mutations examined. MRI data were acquired using various vendors and sequences, including T1C, T2WI, T2-FLAIR, DWI, and DTI. Segmentation methods for extracting radiomic features included manual, semi-automated, and automatic approaches. Modeling methods for analysis encompassed logistic regression, random forest, linear discriminant analysis, and deep learning algorithms (**Figure 1**).

**Table 1:** Characteristics of included studies.

Author	Country	Study period	No. of patients	Age	Gender (M/F)	EGFR mutation rate	Exon of EGFR mutations	MRI vendor	Imaging Sequence	Segmentation method	Modeling method
He, 2019	China	2011-2017	116	≤60: 67, 57.8% >60: 49, 42.2%	82/34	43. 1%	21	Siemens 3.0T	T1C, T2WI, T2-FLAIR	Manual segmentation	ADC histogram analysis
Ahn, 2020	Korea	2012-2018	61	63.2 ± 10.7	36/25	47.5%	19, 20, 21	NR	T1C	Manual segmentation	Random forest
Chen, 2020	USA	2009-2017	110	57.5 ± 12.3	37/73	68.2%	NR	NR	T1C, T2-FLAIR	Semi-automated fashion	Random forest
Park, 2021	Korea	2011-2018	51	59.9 ± 12. 1	26/25	45. 1%	19, 21	Philips 3.0T	DTI, T1C	Semiautomatic method with intensity-based algorithms	Linear discriminant analysis
Wang, 2021	China	2015-2018	52	57.2 ± 9.3	26/26	53.8%	18, 19, 20, 21	Philips 3.0T	T1C, T2WI, T2-FLAIR, DWI	Manual segmentation	Logistic regression
Haim, 2022	Israel, Italy	2009-2019	59	61.9 ± 11.5	33/26	27. 1%	NR	NR	T1WI	Manual segmentation	Deep learning
Jiang, 2022	China	2018-2021	104	63.6 ± 8.3	53/51	57.7%	NR	Siemens 3.0T	3D-T1WI	Manual segmentation	Logistic regression
Bilgin, 2023	Turkey	2017-2021	30	64. 1 ± 11.5	20/10	26.7%	NR	GE 1.5T	T1C, T2WI, T2-FLAIR, DWI	Automatic segmentation	ADC histogram analysis
Fan, 2023	China	2017-2021	230	58.4 ± 9.7	104/126	58.7%	NR	Siemens 3.0T	T1C, T2WI	Manual segmentation	Logistic regression
Li, 2023	China	2015-2022	233	59.6 ± 8.05	86/147	40.8%	20	GE 3.0T	T2-FLAIR, T2WI, T1C, DWI	Manual segmentation	Random forest
Lv, 2023	China	2014-2023	405	56.6 ± 9.8	149/256	37.0%	19, 21	GE 3.0T	T1C, T2WI, T2-FLAIR, DWI	Manual segmentation	Random forest
Zheng, 2023	China	2017-2021	48	63.2 ± 10.4	29/19	47.9%	18, 19, 21	Siemens 3.0T	T1C, T2WI, T2-FLAIR, DWI	Manual segmentation	ADC histogram analysis
Li, 2024	China	2019-2023	399	57.7 ± 10.9	177/222	72.2%	19, 21	GE 3.0T	T2-FLAIR, T1C, T2WI, DWI	Manual segmentation	Deep learning

NR, No reported.

### Quality assessment

**Figure 2:**
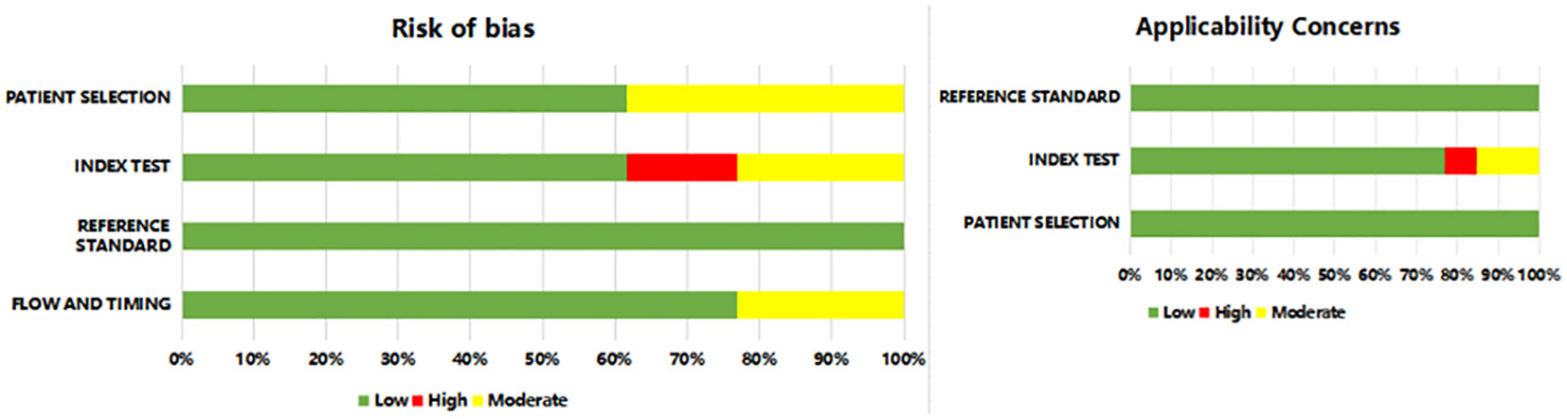
Risk of bias summary.

The methodological quality of the included studies was evaluated using the QUADAS-2 tool. In general, Most studies have a lower risk of bias, and the risk of bias in some studies was still unclear (**Figure 2** and **Supplementary Table S2**). The risk of bias in patient selection was unclear for 5 studies as they were not reported in continuous registration or random sampling. Notably, 2 studies exhibited a high risk of bias in the index test domain, primarily related to the lack of blinding regarding pre-specificity threshold and radiomic approach to the reference standard.

### Synthesis of results and meta-analysis

**Figure 3:**
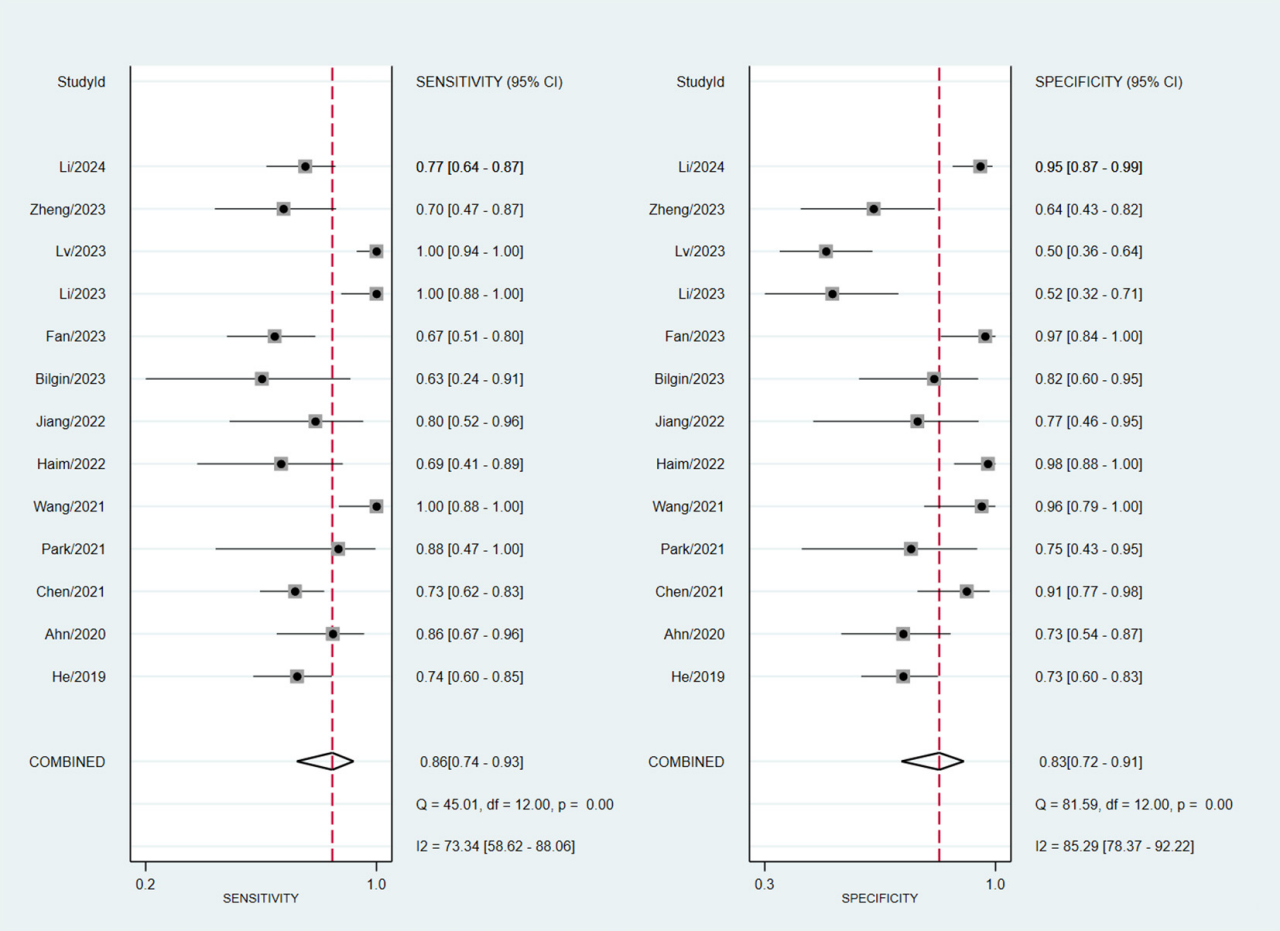
Forest plot of sensitivity and specificity of MRI-based radiomic features of predicting EGFR mutation status in NSCLC patients with brain metastases.

**Figure 4:**
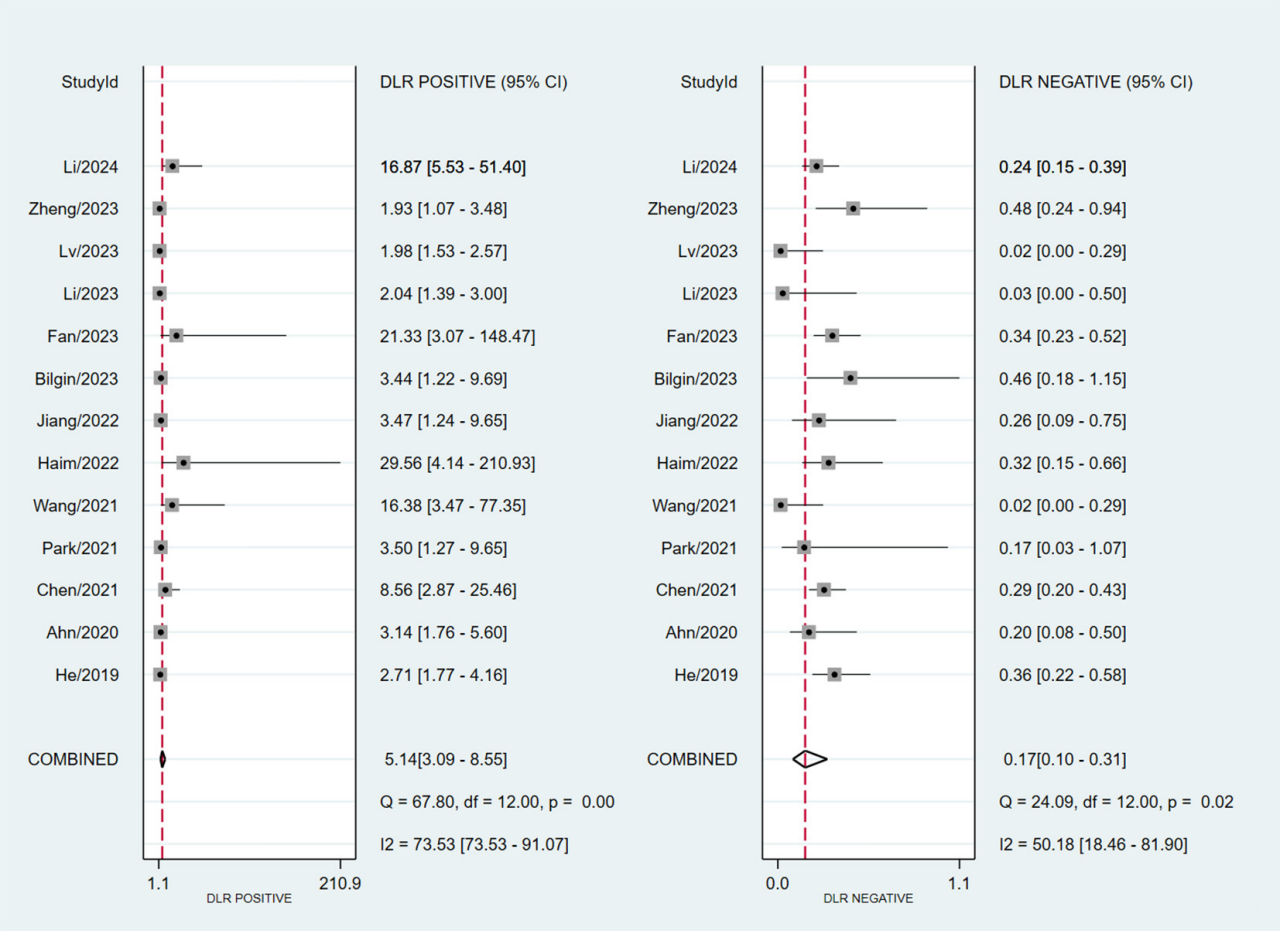
Forest plot of PLR and NLR of MRI-based radiomic features of predicting EGFR mutation status in NSCLC patients with brain metastases.

**Figure 5:**
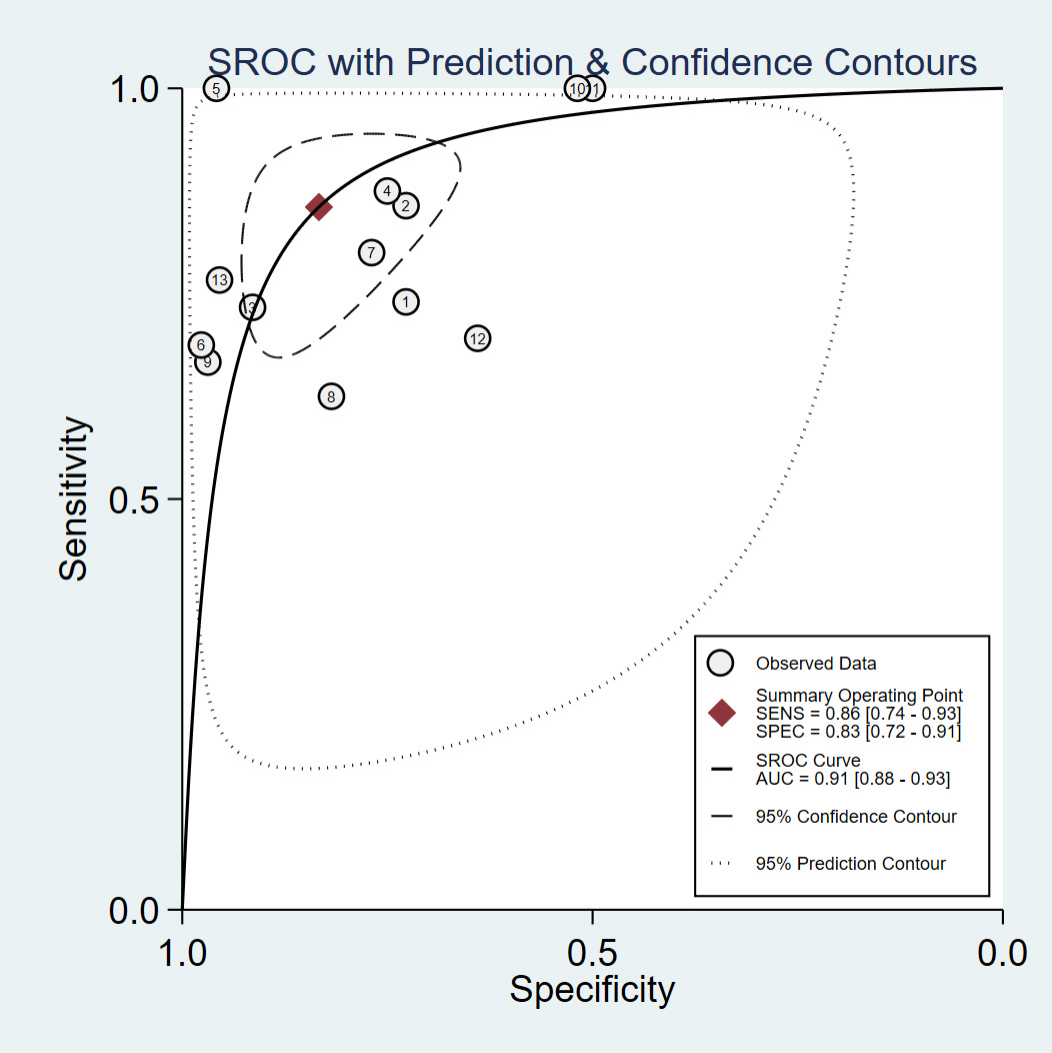
Summary Receiver Operating Characteristic curve for predicting EGFR mutation status in NSCLC patients with brain metastases of MRI-based radiomic features.

Assessing EGFR mutation status on a per-patient basis, overall sensitivity and specificity were 0.86 (0.74, 0.93) and 0.83 (0.72, 0.91), respectively (**Figure 3**). Both plots indicated heterogeneity among the studies (I² = 73.34% for sensitivity and I² = 85.29% for specificity). The PLR and NLR were 5.14 (3.09, 8.55) and 0.17 (0.10, 0.31), respectively (**Figure 4**). The substantial heterogeneity was observed in both panels, with I² values of 73.53% for PLR and 50. 18% for NLR. The AUC was determined to be 0.91, with a CI ranging from 0.88 to 0.93 (**Figure 5**).

### Publication bias

**Figure 6:**
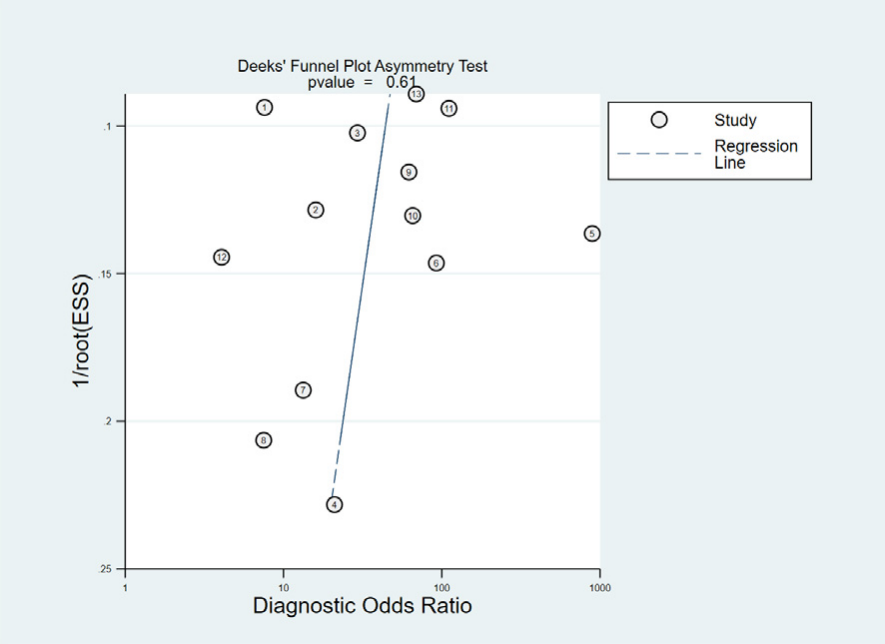
Deek’s Funnel Plot for assessment of Publication bias.

In the included studies, the Deek’s test was used to investigate potential publication bias (**Figure 6**). The plot showed a symmetric distribution around the regression line, suggesting no significant evidence of publication bias across the included studies (p = 0.61).

### Subgroup analyses

**Table 2** detailed the results from the subgroup analysis. The analysis revealed that studies conducted in Asia had higher sensitivity (0.83) but lower specificity (0.75) compared to Euramerican studies, which exhibited lower sensitivity (0.72) and higher specificity (0.92). Automated segmentation strategies resulted in slightly lower sensitivity (0.74) and specificity (0.86) compared to manual segmentation, which achieved a sensitivity of 0.83 and a specificity of 0.78. Studies involving fewer than 100 patients displayed slightly better sensitivity (0.82) and specificity (0.83) than those with more than 100 patients. Deep learning techniques in studies notably achieved higher specificity (0.96) and a higher PLR (19.34) compared to those employing machine learning or ADC histogram analysis.

**Table 2:** Subgroup analysis results.

Analysis	No.	Sensitivity	I^2^, %	Specificity	I^2^, %	PLR	I^2^, %	NLR	I^2^, %	DOR	I^2^, %
Area											
Euramerica	3	0.72	0.0	0.92	59.3	7.73	55.9	0.31	0.0	24.86	35.5
Asia	10	0.83	84.0	0.75	86.0	3.46	78.5	0.26	56.6	24.71	63.1
Segmentation strategy											
Automated	3	0.74	0.0	0.86	12.0	4.60	19.8	0.31	0.0	18.81	0.0
Manual	10	0.83	84.5	0.78	88.8	3.95	82.3	0.27	54.5	28.57	66.0
Number of patients											
< 100	6	0.82	70.6	0.83	76.6	4.21	66.7	0.29	48.9	20.61	64.2
> 100	7	0.80	86.9	0.77	90.1	4.09	85.7	0.28	44.5	28.63	53.9
Modeling method											
Machine learning	8	0.85	86.8	0.74	85.5	3.84	79.4	0.19	67.0	32.25	11.5
Deep learning	2	0.75	0.0	0.96	0.0	19.34	0.0	0.26	0.0	74.26	0.0
ADC histogram analysis	3	0.72	0.0	0.73	0.0	2.50	0.0	0.40	0.0	6.36	0.0

### Clinical utility

**Figure 7:**
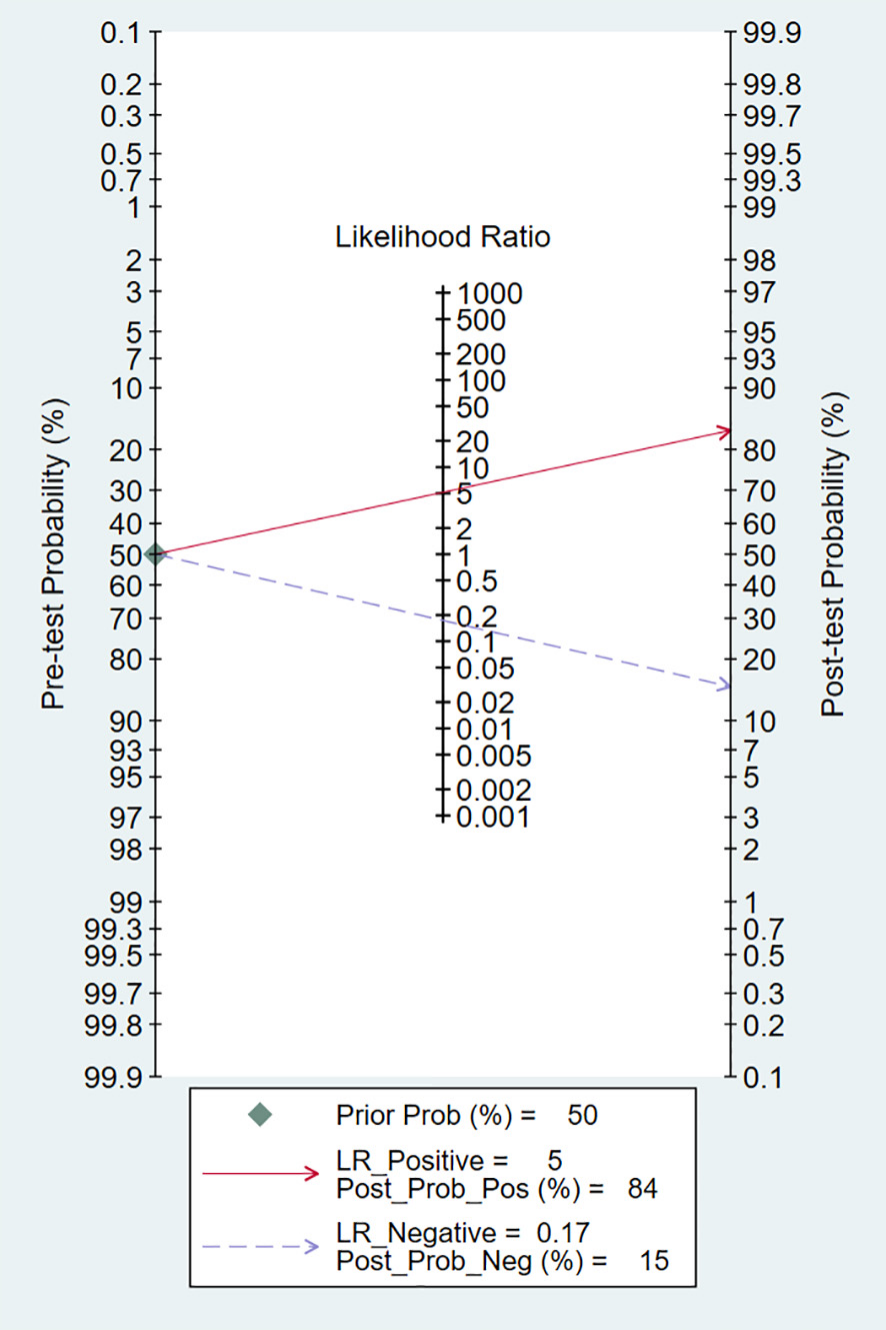
Fagan plots for assessing the clinical utility.

Using an MRI-based radiomic features model would increase the post-test probability to 84% from 50% with a PLR of 5 when the pretest was positive and would reduce the post-test probability to 15% with an NLR of 0.17 when the pretest was negative (**Figure 7**).

## Discussion

To our knowledge, this is the first meta-analysis to evaluate the diagnostic accuracy of MRI-based radiomic features for EGFR mutation status in NSCLC patients with brain metastases. It revealed that while there were substantial differences in sensitivity and specificity based on area, segmentation strategies, number of patients and modeling methods, the overall diagnostic values remained relatively consistent, indicating a robust underlying potential for radiomics in clinical settings. Particularly, deep learning methods emerged as notably effective, providing high specificity and likelihood ratios, which could lead to more precise and reliable diagnostics. However, the pronounced heterogeneity across studies highlighted the critical need for standardized radiomic protocols and uniformity in imaging techniques to enhance the comparability and generalizability of findings.

With the advancements in tumor molecular biology, treatments with epidermal growth factor receptor tyrosine kinase inhibitors (EGFR-TKIs) have provided patients with EGFR mutation-positive advanced lung cancer a longer progression-free survival, especially for patients with brain metastases from EGFR-mutated NSCLC who received erlotinib combined with whole brain radiotherapy (27, 28). Consequently, research into the relationship between EGFR mutation status and radiological features of brain metastases has become a focal point, with efforts to improve qualitative and quantitative diagnosis, differential diagnosis, tumor grading and staging, molecular typing, and evaluation of treatment efficacy in intracranial tumors (29–31).

Various studies have demonstrated a close association between EGFR mutation status and the imaging characteristics of NSCLC patients. CT features of lung adenocarcinoma patients have been shown to correlate with EGFR mutations. Ground-glass opacity (GGO) was frequently observed in patients with EGFR-positive tumors, and the proportion of GGO within the tumor was significantly greater in patients with 21L858R mutations compared to those with 19Del deletions and those with the wild-type gene (32, 33). A meta-analysis from France further provided a thorough evaluation of CT radiomics-based models for predicting EGFR mutation status in patients with NSCLC, with a pooled AUC of 0.801 (34). In PET/CT scans, the status of EGFR mutations was associated with lower SUVmax values (35). The SUVmax of pulmonary lesions served as a predictive marker for EGFR mutations, whereas metabolic tumor volume and total lesion glycolysis were not related to EGFR mutation status (36). However, it was reported that ^18^F-FDG PET/CT radiomic features have significant potential in predicting EGFR mutation subtypes in patients with lung adenocarcinoma, achieving a notable AUC of 0.87 for the combined model of predicting EGFR mutation positivity (37). A meta-analysis that encompassed 17 studies suggested that 18F-FDG PET/CT radiomics could assist in predicting EGFR gene mutation status in NSCLC patients, achieving a pooled AUC of 0.82 (38). Additionally, there have been increasing reports on the correlation between MRI imaging characteristics and EGFR mutation status in patients with brain metastases from NSCLC (39). Specifically, patients with the 19Del mutation have tended to exhibit smaller brain metastases and peritumoral edema, as well as a higher number of lesions (40). It was also revealed that lesions in patients with 21L858R mutation more frequently occur in specific regions such as the caudate nucleus, cerebellum, and temporal lobes, with brain lesions in 21L858R mutation group more superficial compared to those in the 19Del and wild-type groups (41).

AI-driven technologies, particularly in radiomics and pathomics, enhanced diagnosis, treatment decisions, and prognostic assessments in NSCLC by predicting oncogenic driver mutations and immune checkpoint inhibition responses (42). Random forest (RF) and convolutional neural network (CNN) models based on CT images were utilized to differentiate between the EGFR mutation subtypes (wild-type, 19Del and 21L858R), effectively achieving satisfactory AUCs both training and validation sets (43, 44). Besides, machine learning techniques in the field of MRI radiomics was applied to predict survival durations in patients with brain metastases from NSCLC, exhibiting impressive diagnostic accuracies with AUCs of more than 90% for all EGFR, ALK, and KRAS mutation-positive groups (16). Furthermore, Li et al. developed graph convolutional network (Radio-GCN) model to predict EGFR mutation subtypes using multisequence MRI, achieving area AUCs of 0.996, 0.971, and 1.000 for identifying EGFR 19Del, 21L858R, and wild-type statuses respectively (26).

However, the challenges of applying radiomics in heterogeneous clinical scenarios like brain metastases should not be ignored. For example, while the T2-FLAIR sequence showed promising results with an AUC of 0.987 for predicting EGFR mutation status and 0.871 for validating in the independent testing dataset, had inadequate performance in distinguishing between 19Del and 21L858R mutation subtypes (18). Based on the significant potential of predicting EGFR mutation status, future research should focus on validating these models across larger, multi-center cohorts to confirm their generalizability. Additionally, integrating radiomic features with other molecular or genetic biomarkers could enhance diagnostic precision (45). Exploring the real-time application of these models in clinical settings and employing advanced machine learning techniques could further refine their effectiveness and provide insights into tumor evolution and therapy responses.

Our study aggregated data from multiple studies, which increased the statistical power and generalizability of the findings in this field. However, there were several limitations. First, variations in radiomic methodologies, imaging protocols, and patient selection criteria across the included studies introduced heterogeneity, complicating the aggregation and interpretation of results. Second, there was a potential for publication bias, as studies with positive findings were more likely to be published, which could have skewed the overall analysis results. Third, restricting the inclusion to studies published in English and Chinese might have excluded relevant data from other populations and settings. Fourth, the absence of individual patient data limited the ability to perform detailed subgroup analyses and may have reduced the precision of our findings, affecting the ability to tailor results to specific patient groups.

## Conclusions

Our meta-analysis has demonstrated the significant potential of MRI-based radiomic features for accurately predicting EGFR mutation status in NSCLC patients with brain metastases. These findings suggest that radiomics could play a pivotal role in the non-invasive diagnosis and personalized treatment planning for these patients, potentially leading to more tailored and effective therapeutic approaches. By incorporating advanced imaging techniques into clinical practice, healthcare providers can enhance decision-making processes, optimize treatment strategies, and improve patient outcomes in oncology.

## Data Availability

The datasets generated during and/or analysed during the current study are available from the corresponding author on reasonable request.
