# Supplementary material for: Diagnostic accuracy of MRI-based radiomic features for EGFR mutation status in non-small cell lung cancer patients with brain metastases: a meta-analysis
Source: Front Oncol. 2025 Jan 6;14:1428929. doi: 10.3389/fonc.2024.1428929 (PMC11743156; doi:10.3389/fonc.2024.1428929)
Supplement: Supplementary file 1 [file Table1.docx]

**Supplementary information**

Table S1. Search Strategy

| Database | Search Entry |
| --- | --- |
| PubMed | ("non-small cell lung cancer" OR "NSCLC") AND ("brain metastases" OR "brain neoplasm") AND ("EGFR mutation" OR "epidermal growth factor receptor mutation") AND ("MRI" OR "magnetic resonance imaging") Result: 32 |
| Embase | ('non-small cell lung cancer'/exp OR 'NSCLC'/exp) AND ('brain metastasis'/exp OR 'brain neoplasm'/exp) AND ('EGFR mutation'/exp OR 'epidermal growth factor receptor mutation'/exp) AND ('MRI'/exp OR 'magnetic resonance imaging'/exp) Result: 30 |
| Cochrane Library | (("non-small cell lung cancer" OR NSCLC) AND ("brain metastases" OR "brain neoplasm") AND ("EGFR mutation" OR "epidermal growth factor receptor mutation") AND ("MRI" OR "magnetic resonance imaging")) NOT (conference abstracts OR case reports OR editorials) in All Text Result: 12 |
| China National Knowledge Infrastructure (CNKI) | ("non-small cell lung cancer" + "NSCLC") AND ("brain metastases" + "brain neoplasm") AND ("EGFR mutation" + "epidermal growth factor receptor mutation") AND ("MRI" OR "magnetic resonance imaging") Result: 523 |
| Wanfang | ("non-small cell lung cancer" OR "NSCLC") AND ("brain metastases" OR "brain neoplasm") AND ("EGFR mutation" OR "epidermal growth factor receptor mutation") AND ("MRI" OR "magnetic resonance imaging") Result: 38 |
| Web of Science | non-small cell lung cancer OR NSCLC (All Fields) and brain metastases OR brain neoplasm (All Fields) and EGFR mutation OR epidermal growth factor receptor mutation (All Fields) and MRI OR magnetic resonance imaging (All Fields) Result: 98 |
| Scopus | ("non-small cell lung cancer" OR "NSCLC") AND ("brain metastases" OR "brain neoplasm") AND ("EGFR mutation" OR "epidermal growth factor receptor mutation") AND ("MRI" OR "magnetic resonance imaging") in Title, abstract, key word Result: 224 |
