# Supplementary material for: Diagnostic accuracy of MRI-based radiomic features for EGFR mutation status in non-small cell lung cancer patients with brain metastases: a meta-analysis
Source: Front Oncol. 2025 Jan 6;14:1428929. doi: 10.3389/fonc.2024.1428929 (PMC11743156; doi:10.3389/fonc.2024.1428929)
Supplement: Supplementary file 2 [file Table2.docx]

**Table S2. Quality Assessment of each study**

|  | **Risk of Bias** | | | | **Applicability Concerns** | | |
| --- | --- | --- | --- | --- | --- | --- | --- |
| **Study ID** | **PATIENT SELECTION** | **INDEX TEST** | **REFERENCE STANDARD** | **FLOW AND TIMING** | **PATIENT SELECTION** | **INDEX TEST** | **REFERENCE STANDARD** |
| He, 2019 | Unclear | High | Low | Unclear | Low | High | Low |
| Ahn, 2020 | Unclear | Low | Low | Low | Low | Low | Low |
| Chen, 2020 | Low | Unclear | Low | Low | Low | Low | Low |
| Park, 2021 | Low | Low | Low | Low | Low | Low | Low |
| Wang, 2021 | Low | Low | Low | Unclear | Low | Unclear | Low |
| Haim, 2022 | Unclear | Unclear | Low | Low | Low | Low | Low |
| Jiang, 2022 | Unclear | High | Low | Unclear | Low | Unclear | Low |
| Bilgin, 2023 | Low | Low | Low | Low | Low | Low | Low |
| Fan, 2023 | Low | Low | Low | Low | Low | Low | Low |
| Li, 2023 | Low | Unclear | Low | Low | Low | Low | Low |
| Lv, 2023 | Low | Low | Low | Low | Low | Low | Low |
| Zheng, 2023 | Unclear | Low | Low | Low | Low | Low | Low |
| Li, 2024 | Low | Low | Low | Low | Low | Low | Low |
